# Participant experience in observational studies of brain aging and Alzheimer's disease

**DOI:** 10.1002/alz70858_106749

**Published:** 2025-12-26

**Authors:** Amber Ligason‐Tiquia, Maricarmen Pachicano, Elena Taylor‐Munoz, Helena C Chui, Elizabeth B Joe

**Affiliations:** ^1^ Western University of Health Sciences‐ College of Osteopathic Medicine of the Pacific, Pomona, CA, USA; ^2^ USC Center for Integrative Connectomics, Los Angeles, CA, USA; ^3^ Keck School of Medicine, University of Southern California, Los Angeles, CA, USA; ^4^ Alzheimer's Disease Research Center, Keck School of Medicine, University of Southern California, Los Angeles, CA, USA; ^5^ Department of Neurology, Keck School of Medicine, University of Southern California, Los Angeles, CA, USA

## Abstract

**Background:**

Longitudinal studies of aging and cognition depend on high rates of participant retention. We conducted surveys of participants in observational research studies through USC's Alzheimer's Disease Research Center (ADRC) to assess their overall experience with research involvement.

**Method:**

3 waves of participant feedback surveys were conducted over a 4 year period. Surveys were mailed to English‐speaking participants without dementia enrolled in the ADRC and affiliated longitudinal biomarker‐based studies. Respondents completed surveys asking them to describe their experience with study staff, participation logistics, and individual research procedures. Two raters coded free text responses thematically and discrepancies were reconciled by consensus.

**Result:**

Results from 222 surveys were analyzed. Key themes associated with a positive experience with research participation were staff qualities, such as professionalism and warmth, and a sense of partnership with the research team. Negative comments primarily related to staff turnover, communication, inconvenience, and discomfort from study procedures, such as MRI noise or spinal headache after lumbar puncture. In addition, some responses reflected a desire for more streamlined disclosure of research results, or demonstrated a lack of clarity of the relationship between research and clinical care.

**Conclusion:**

While most respondents had positive experiences with research participation, a number of modifiable factors were associated with negative participant experience. Identifying facilitators and barriers of research participation may improve participant retention in longitudinal studies of aging research.

